# Scalable high-repetition-rate sub-half-cycle terahertz pulses from spatially indirect interband transitions

**DOI:** 10.1038/s41377-022-00824-6

**Published:** 2022-05-23

**Authors:** Christian Meineke, Michael Prager, Johannes Hayes, Qiannan Wen, Lukas Zheyi Kastner, Dieter Schuh, Kilian Fritsch, Oleg Pronin, Markus Stein, Felix Schäfer, Sangam Chatterjee, Mackillo Kira, Rupert Huber, Dominique Bougeard

**Affiliations:** 1 Department of Physics, University of Regensburg, 93040 Regensburg, Germany; 2 Department of Electrical Engineering and Computer Science, University of Michigan, Ann Arbor, MI 48109 USA; 3 Faculty of Electrical Engineering, Helmut Schmidt University, 22043 Hamburg, Germany; 4 Institute of Experimental Physics I, Justus Liebig University Giessen, 35392 Giessen, Germany

**Keywords:** Ultrafast lasers, Mid-infrared photonics, Terahertz optics, Nonlinear optics

## Abstract

Intense phase-locked terahertz (THz) pulses are the bedrock of THz lightwave electronics, where the carrier field creates a transient bias to control electrons on sub-cycle time scales. Key applications such as THz scanning tunnelling microscopy or electronic devices operating at optical clock rates call for ultimately short, almost unipolar waveforms, at megahertz (MHz) repetition rates. Here, we present a flexible and scalable scheme for the generation of strong phase-locked THz pulses based on shift currents in type-II-aligned epitaxial semiconductor heterostructures. The measured THz waveforms exhibit only 0.45 optical cycles at their centre frequency within the full width at half maximum of the intensity envelope, peak fields above 1.1 kV cm^−1^ and spectral components up to the mid-infrared, at a repetition rate of 4 MHz. The only positive half-cycle of this waveform exceeds all negative half-cycles by almost four times, which is unexpected from shift currents alone. Our detailed analysis reveals that local charging dynamics induces the pronounced positive THz-emission peak as electrons and holes approach charge neutrality after separation by the optical pump pulse, also enabling ultrabroadband operation. Our unipolar emitters mark a milestone for flexibly scalable, next-generation high-repetition-rate sources of intense and strongly asymmetric electric field transients.

## Abstract (web summary)

We generate extremely asymmetric sub-half-cycle terahertz waveforms with scalable field strengths at megahertz pulse-repetition rates via ultrafast excitation of spatially indirect interband transitions in tailored quantum well heterostructures.

## Graphical abstract

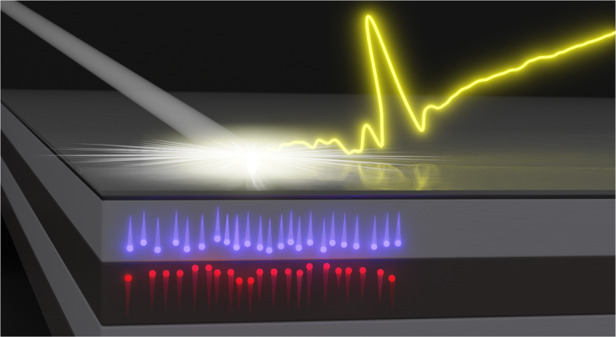

## Introduction

Ultrashort pulses in the terahertz (THz) spectral range represent the most direct tools to probe and control low-energy elementary dynamics in condensed matter^1–4^. Recently, intense phase-locked THz waveforms with octave-spanning spectra and sub-cycle durations have enabled the advent of THz lightwave electronics, where the strong carrier field serves as a transient bias to drive ultrafast currents^5–12^. Tailored THz fields have been employed to open sequential tunnelling channels in stationary junctions^13^ or operational scanning tunnelling microscopes, in time windows much shorter than a single oscillation period of the carrier wave^14–19^. In all these applications, unidirectional currents would be ideally driven by hypothetical strictly unipolar THz waveforms made of a single oscillation half-cycle. Since electromagnetic waves propagating in the far field are expected to require AC fields^20^, however, the best possible THz waveforms consist of asymmetric bipolar transients in which a dominant positive half-cycle dramatically exceeds the strength of feeble negative excursions needed to cancel the temporal integral of the electric field. Moreover, practical lightwave electronic experiments and future device applications demand large and scalable field strengths (typically 1 kV cm^–1^ and higher) combined with high repetition rates of 1 MHz and above to drive the required nonlinearities and guarantee competitive signal-to-noise ratios.

Most sources aiming to meet these criteria are based on frequency conversion of near-infrared (NIR) femtosecond laser pulses. Difference frequency mixing via *χ*^(2)^ nonlinearities in non-centrosymmetric media has provided intense sub-cycle THz pulses^21,22^. However, upscaling their field strength, e.g., by increasing the length of the nonlinear crystal, limits the bandwidth. Photocurrents in transient gas plasmas give rise to multi-octave-spanning spectra^23–25^, but the necessary pump-pulse energies exceeding 0.1 mJ limit this technique to relatively low-repetition-rate lasers. Emitters based on spin-to-charge current conversion via the inverse spin-Hall effect have marked another important breakthrough as these metal-based spintronic THz emitters generate octave-spanning, sub-cycle THz waveforms^26–29^. The finite optical skin depth of metals prevents the thickness scaling of these emitters such that high THz field strengths have required large pump-pulse energies. Recently, a promising alternative mechanism generating ultrashort THz transients was observed in van der Waals heterostructures. After optical excitation of electron–hole pairs in a heterobilayer of transition metal dichalcogenides (TMDCs) featuring type-II band alignment, charge separation by shift currents has given rise to a sub-cycle THz pulse^30,31^. However, the efficiency of this scheme is rather low as the charge separation length is small. Furthermore, these emitters are only as scalable as the delicate stacking of TMDC monolayers permits. Lastly, the inherently fixed resonances and the complicated simulation of the microscopic processes causing charge separation make it tough to optimise and custom-tailor the THz emission. In contrast, band-gap engineering in semiconductor quantum wells (QWs) allows matching electronic transition energies to a pump source of choice. This has been employed to generate few- and sub-cycle THz pulses via photoexcitation of electron–hole pairs in DC-biased QWs^32,33^ and intersubband transitions in asymmetric QWs^34^, but has not been scaled to high-power pump lasers.

Here we transfer the concept of shift-current-based THz generation in type-II aligned nanostructures to epitaxial semiconductor heterostructures and introduce a fully scalable THz source capable of generating strongly asymmetric sub-cycle field transients. The key idea is to engineer electronic wavefunctions in asymmetrically coupled semiconductor QWs such that resonant interband photoexcitation induces an ultrafast charge separation by shift currents over several nanometres even without any bias. By fine-tuning the interband transitions to the spectral range of state-of-the-art high-power ytterbium fibre pump lasers, we generate strong THz pulses featuring only 0.45 optical cycles at their centre frequency within the full width at half maximum (FWHM) of their intensity envelope at repetition rates up to 4 MHz. By tuning the pump-pulse spectrum and duration, we can achieve a positive field maximum of up to 1.1 kV cm^–1^, which exceeds the strongest negative excursions by a factor of 3.7. Owing to the lattice-matched unstrained growth, the emitter concept is scalable to yet higher field strengths by straightforwardly increasing the number of growth repetitions.

## Results

**Fig. 1:**
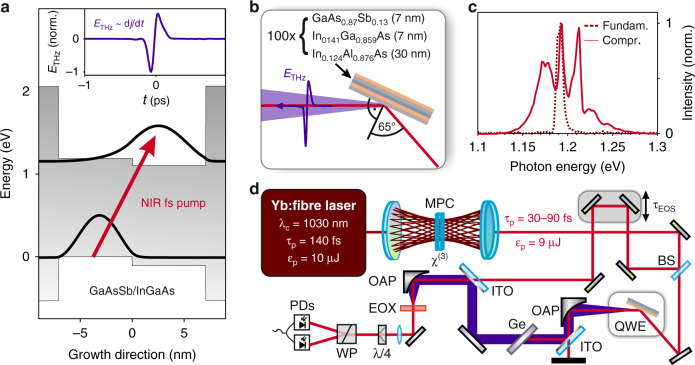
Concept and experimental setup of THz generation from quantum well emitters. **a** When an intense NIR femtosecond light pulse (red arrow) generates electron–hole pairs in the highest hole subband and the lowest electron subband (black) of a type-II aligned QW, an ultrafast dipole moment arises owing to direct excitation of spatially separated electron–hole pairs. Inset: a mostly symmetric, sine-shaped THz waveform is expected. **b** The QWE consisting of 100 GaAs_0.87_Sb_0.13_/In_0.141_Ga_0.859_As QWs separated by In_0.124_Al_0.876_As barriers is pumped in reflection geometry under an angle of incidence of 65°. **c** Spectral intensity of the fundamental (dark red dotted line) and compressed (red solid line) laser pulses. **d** Femtosecond pulses of an ytterbium fibre laser are spectrally broadened and compressed to 30–90 fs using self-phase modulation in a fused silica medium (*χ*^(3)^) in a multi-pass cell (MPC). The pulses act both as a pump for the quantum well emitters and as a gate in the electro-optic detection using a GaSe crystal with a thickness of 6 µm as electro-optic crystal (EOX). BS beam splitter, τ_EOS_ delay time, OAP off-axis parabolic mirror, ITO fused silica window with one-sided indium tin oxide coating, Ge 500-µm-thick germanium window, λ/4 quarter-wave plate, WP Wollaston prism, PDs balanced photodiodes

The idea of the THz emitter is sketched in Fig. 1a. Two semiconductor QWs with type-II band alignment are embedded in potential barriers such that the lowest conduction subband (black) can be populated via resonant interband excitation (red arrow). Since the envelope functions of the highest-energy valence subband (black) and the lowest conduction subband are concentrated on opposite halves of the confinement potential, optical excitation induces an ultrafast charge separation. Unlike in schemes exploiting in-plane currents^35,36^, carrier diffusion plays no prominent role here because the out-of-plane motion is fully quantised. Thus, in a simplistic picture, where the photoexcited electrons and holes move in a static potential landscape, femtosecond interband excitation may then lead to an ultrashort unidirectional shift-current burst, which emits THz radiation according to Maxwell’s equations. Since the THz far field follows the time derivative of the current, a strict single-cycle waveform with equally strong positive and negative peaks is expected (see Fig. 1a, inset, and Supplementary Fig. S2).

We test the potential of this scenario with a suitable heterostructure based on In_*x*_Ga_1–*x*_As and GaAs_1–*x*_Sb_*x*_ type-II-QWs with In_*x*_Al_1–*x*_As potential barriers. By controlling the material composition and the thickness of QWs and barriers with atomic precision, the band gaps, band offsets, confinement energy, subband energies, and spatial distribution of the subband states are precisely set. We tune the optical interband transitions into resonance with the output spectrum of state-of-the-art commercial ytterbium fibre pump lasers. The envelope functions of the frontier valence and conduction subband states are spatially separated by as much as six nanometres, allowing for giant transient dipole moments upon photoexcitation without any external bias. All elements of the heterostructure are fully lattice-matched, which allows for stacking of multiple active QW units. Our QW emitter (QWE) contains 100 repetitions of GaAs_0.87_Sb_0.13_(7 nm)/In_0.141_Ga_0.859_As(7 nm) QWs separated by In_0.124_Al_0.876_As barriers. As the expected photoinduced shift currents are directed parallel to the growth direction, so is the polarisation of the emerging THz dipole radiation. In reflection geometry, it constructively interferes along the direction of the specular reflection of the pump beam (Fig. 1b). We choose a pump angle of incidence of 65°, close to Brewster’s angle of the NIR pump, to simultaneously maximise the THz intensity retrieved from the inherent dipole-like radiation pattern while minimising the reflection losses of the NIR pump pulses and the generated THz pulses exiting the QWE.

Intense pump pulses are received from an ytterbium fibre laser (centre wavelength 1030 nm, pulse duration 140 fs, pulse energy 10 µJ, repetition rate 4 MHz). Using self-phase modulation in fused silica placed inside a multi-pass cell^37^ (Fig. 1d), the fundamental laser spectrum (Fig. 1c, red dashed line) can be broadened, compressing the pulse in time. By moving the nonlinear medium relative to the focal position, the pulse duration can be continuously tuned. The broadest achievable spectrum (Fig. 1c, red solid line) contains photon energies (frequencies) between 1.13 eV (273 THz) and 1.28 eV (309 THz), features a FWHM of 50 meV (12 THz) and is centred at 1.2 eV (291 THz). The nearly bandwidth-limited compressed laser pulse has a duration of 30 fs (FWHM) and a pulse energy of 9 µJ. We split the compressed pulse into a stronger pump and a weaker gate. When the QWE is pumped at a fluence of 1 mJ cm^−2^, intense, phase-stable THz field transients are generated, collimated by an off-axis parabolic mirror (8” focal length) and refocused by another parabolic mirror (2” focal length) into a gallium selenide crystal (thickness, 6 µm) acting as electro-optic crystal^38^. There, the THz pulses are spatially and temporally overlapped with the gate pulses to electro-optically resolve the electric carrier field of the THz pulse as a function of the delay time *t*. In our measurements, we take special care to ensure that the detector is placed in the THz focus to avoid artefacts owing to the Gouy phase shift^39^ (see Supplementary Fig. S5).

**Fig. 2:**
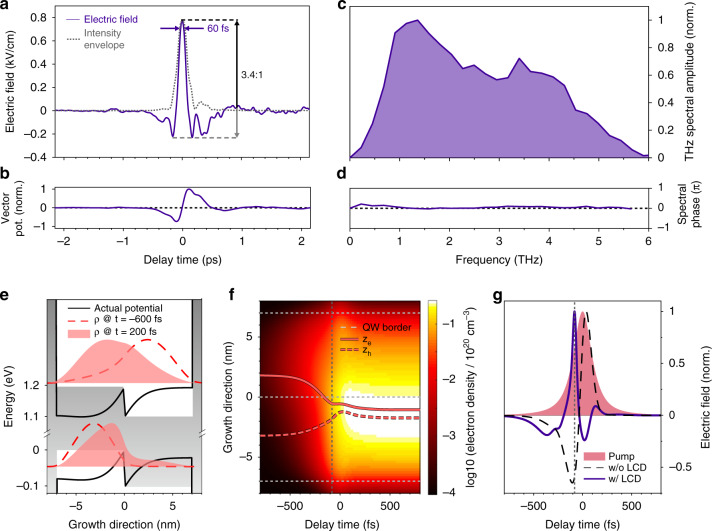
Unipolar THz transients and full quantum theory–experiment comparison. **a** THz electric field waveform (purple) detected via electro-optic sampling in a 6-µm-thick GaSe crystal and corrected for the detector response. The intensity envelope (grey dotted line) has a FWHM duration of 183 fs, which corresponds to 0.45 optical cycles at the centre frequency of 2.47 THz. The waveform features an extreme asymmetry ratio of 3.4:1 with a peak electric field strength of 0.77 kV cm^–1^. **b** The vector potential vanishes at large negative and positive delay times. **c** Octave-spanning spectrum of the THz pulse including spectral components up to 6 THz. **d** Flat spectral phase across all spectral components with a relative amplitude of 10% or more. **e** Computed initial QW confinement (grey area) and carrier density (red dashed lines) *t* = –600 fs before the pump pulse with a duration of 130 fs. Local charging dynamics (LCD) is fully included to produce the actual potential at *t* = 200 fs after the pump pulse (black lines) and the corresponding carrier density (red shaded areas). **f** Colour-coded plot of the computed electron density as a function of time along the growth direction. Red solid (dotted) line identifies the mean electron (hole) position as a function of delay time; horizontal grey dotted lines denote the QW interfaces and the vertical grey dotted line the time of the unipolar emission peak. **g** Pump field amplitude (red shaded area), and theoretically predicted THz waveform with (solid purple) and without (dashed-black line) LCD

The detected THz transient, generated with a pump-pulse duration of 70 fs, and corrected for the detector response (see Supplementary Fig. S3), follows a surprising waveform (Fig. 2a, purple), which differs qualitatively from the shape anticipated in Fig. 1a: an extremely sharp single maximum at *t* = 0 ps reaching a field strength of 0.77 kV cm^−1^ dominates over two weak negative excursions at *t* = −0.16 ps (electric field, 0.22 kV cm^−1^) and *t* = 0.17 ps (electric field, 0.23 kV cm^−1^). Comparing the field strengths of the largest half-cycles of opposite signs, we receive an asymmetry ratio of 3.4:1. Despite the extreme asymmetry, the integral over the electric field should vanish for a far-field electromagnetic wave propagating in free space^20^. This is equivalent to a vanishing vector potential at infinite positive and negative delay times, which we actually observe for the measured field transient (Fig. 2b).

At its centre frequency of 2.47 THz, the pulse features only 0.45 optical cycles within the FWHM duration of the intensity envelope of 183 fs (Fig. 2a, dotted grey line, see Supplementary Note 4 for details). Many lightwave-electronics experiments necessitate extremely short main maxima; typically, the temporal width Δτ of the field crest at 90% of the maximum field strength is the main figure of merit. This field crest can, e.g., open a tunnelling time window in a scanning tunnelling microscope^15^. For our pulses, this window has a duration of only Δτ = 60 fs. The spectral amplitude retrieved by Fourier transform (Fig. 2c) covers more than 2.6 optical octaves with its FWHM ranging from 0.68 to 4.3 THz. It peaks at 1.36 THz and features spectral components up to 6 THz. The spectral phase is flat over the entire range where the spectral amplitude is larger than 10% indicating that the pulse is Fourier-limited (Fig. 2d).

## Discussion

The extremely asymmetric shape of the measured THz transient (Fig. 2a) stands in stark contrast to the simplistic model transient of Fig. 1a. To resolve this discrepancy, we self-consistently solve the wave equation, the semiconductor Bloch equations^40^, and local charging dynamics (LCD). This allows us to systematically describe THz emission, optical excitations, shift currents, and Coulombic many-body dynamics among photoexcited electrons and holes as well as spatially separated charges for the experimental QW system. By generalising the approach of ref. ^41^ for quantum-confined systems, we not only solve how shift currents alter the QW confinement levels but also determine the resulting LCD effects. Figure 2e compares initial (*t* = −600 fs, grey-shaded areas) and final (*t* = 200 fs, black lines) confinement potentials for the lowest electron (top) and hole (bottom) subbands after optical excitation by a pulse with a duration of 130 fs; also the corresponding initial (red dashed lines) and final (red shaded areas) carrier confinement functions are shown.

Including LCD yields massive changes to the electron–hole distribution. This is particularly clear in Fig. 2f, which presents the full evolution of the total electron density (colour map) together with the mean electron (red solid line) and hole (red dashed line) positions along the confinement direction. While the photoexcited electrons and holes are initially separated by 5 nm, as assumed in the simplified analysis of Fig. 1a, the LCD pulls the electrons and holes back together, yielding an abrupt decrease in the electron–hole separation, around *t* = 0. Figure 2g compares this sudden dipole switching (vertical lines in Fig. 2f, g) with THz emission for a 130 fs excitation pulse (shaded area). Specifically, only the computation with LCD (blue-solid line) produces a pronounced unipolar THz-emission peak synchronised with dipole switching, whereas the computation without LCD (dashed line) results in the same bipolar emission as predicted in Fig. 1a. We find that the large positive peak emerges when the electron–hole pairs transiently approach charge neutrality, while the negative emission peaks result from relaxation oscillations around the charge-neutral quasi-equilibrium. Hence, the measured asymmetric waveform originates from a new mechanism where an intriguing interplay of shift currents and LCD switches the dipole to compensate optically generated local charging.

**Fig. 3:**
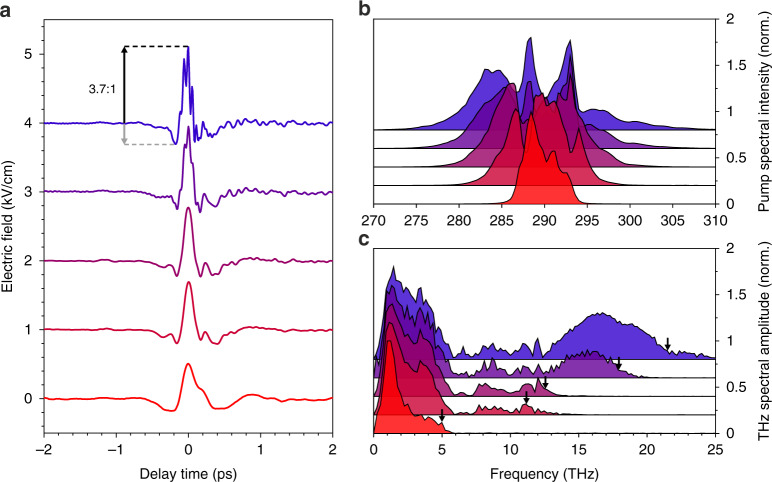
Scaling the THz bandwidth. **a** THz waveforms for different pump-pulse durations and spectra. From red to blue the pump-pulse duration changes from 140 to 30 fs. **b** Corresponding pump spectra. **c** Normalised THz spectral amplitude of the respective waveforms shown in panel **a**. The highest measured THz frequencies (10% of the maximum spectral amplitude) are indicated by black arrows

Importantly, unlike in coupled QWs separated by a finite tunnelling barrier^42^, this mechanism does not require inherently bandwidth-limiting scattering processes to spatially separate photoexcited electrons and holes. Therefore, our THz pulses should be flexibly adjustable by the pump duration. To test this experimentally, we gradually increase the pump spectral bandwidth from 3.6 THz (full width at tenth maximum [FWTM]) up to 22.3 THz FWTM (Fig. 3b) and simultaneously reduce the duration of our pump pulses from 140 fs down to 30 fs. The pump fluence remains fixed at 1 mJ cm^−2^. Figure 3a shows the detected THz waveforms after correction for the detector response. Irrespective of the pump spectrum, all transients maintain a strongly asymmetric shape where the asymmetry ratio increases from 2.8 (lowest, red transient) at the narrowest pump spectrum to 3.7 (highest, blue transient) at the most broadband pump spectrum, even exceeding the asymmetry of the waveform shown in Fig. 2a. This result agrees with our microscopic theory, where decreasing the pump-pulse duration accelerates the LCD, which causes the strong positive oscillation half-cycle of the THz field. Thus, using shorter pump pulses defines an effective strategy to maximise the asymmetry of our THz transients (see also Supplementary Note 5).

Furthermore, reducing the pump-pulse duration monotonically increases the peak field of the THz transients (Fig. 3a) from 0.51 up to 1.1 kV cm^−1^, while the width of the field crest Δτ is reduced from 70 fs down to only 20 fs. The significant decrease of Δτ can be attributed to high THz frequency components that emerge at large pump bandwidths. The corresponding THz spectra are shown in Fig. 3c. We see that when the pump-pulse bandwidth is increased, so is the highest THz Fourier component. Although the THz spectra are marked by phonon absorption in the emitter structure around 8 THz, notably, the FWTM of the pump spectrum strongly correlates with the highest measured THz frequency: pump FWTMs of 7.3, 13.2, 17.4, 18.8, and 22.3 THz (Fig. 3b) lead to spectral THz components of up to 5, 11.2, 12.2, 17.9, and 21.4 THz (Fig. 3c, black arrows), respectively. This correlation indicates that the THz bandwidth of our QWEs can be controlled via the pump-pulse duration and could even be further increased by pumping with shorter NIR pulses.

**Fig. 4:**
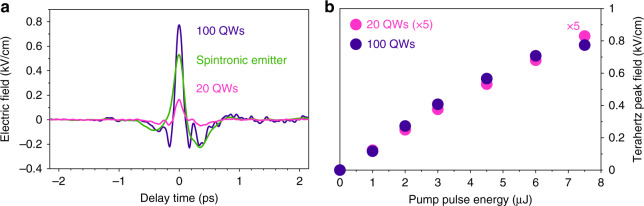
Scalability of the emission scheme. **a** Comparison of the electric field waveforms generated in two QWEs and a spintronic emitter. Scaling the number of QWs from 20 (black) to 100 (purple) increases the field strength by a factor of 4.7. With field strengths of 0.77 kV cm^–1^ at MHz repetition rates, the peak field of spintronic emitters (green) is surpassed. **b** Scaling of the THz peak field with the incident pump-pulse energy. The peak field of the emitter with 20 QWs (black) is multiplied by a factor of 5 to allow for a better comparison with the emitter with 100 QWs (purple). The peak field of both emitters scales linearly with the pump-pulse energy until saturation sets in at very high intensities

Beyond the almost freely extendable bandwidth of our QWE generation scheme, the THz power should be scalable as well. To assess the scalability of the field strength of the QWEs, we compare the field transients from QWEs consisting of 20 and 100 QWs, respectively. At a pump-pulse duration of 70 fs, we observe an increase of the peak electric field by a factor of 4.7, when the number of QWs is increased from 20 to 100 (Fig. 4a). The general shape of the waveform remains unchanged. With 100 QWs, a peak field of 0.77 kV cm^–1^ was reached at a pulse-repetition rate of 4 MHz, even surpassing the field strength of 0.53 kV cm^–1^ generated in a benchmark spintronic emitter^27^, composed of a W(2 nm)/Co_40_Fe_40_B_20_(1.8 nm)/Pt(2 nm) trilayer, under identical conditions. By further increasing the number of QWs, even higher field strengths could come into reach. Furthermore, the higher asymmetry ratio of 3.4:1 compared to 2.3:1 of spintronic emitters makes these pulses particularly attractive for many applications^13–19^. Extending this scheme to higher pump-pulse energies could further enhance the emitted THz peak field. As shown in Fig. 4b, the peak field scales linearly with the pump-pulse energy. Above a pump-pulse energy of 7 µJ, first signs of saturation are visible, which can be overcome by increasing the pump-beam diameter to reduce the pump fluence. Owing to the high thermal conductivity of GaAs, these emitters promise functionality at even higher pulse-repetition rates and pump-pulse energies.

In conclusion, we have developed a novel scalable THz-emitter concept based on an ultrafast interplay of shift currents and LCD in semiconductor QW heterostructures. Electronic wavefunctions in type-II aligned QWs are tailored such that resonant interband photoexcitation by a state-of-the-art, high-power femtosecond laser drives ultrafast shift currents generating 0.45-cycle THz waveforms. The outstanding asymmetry ratio of up to 3.7:1 originates from a new mechanism where the shift-current-induced dipole is abruptly quenched by LCD. Exploiting the scalability of this scheme, we generated THz waveforms with spectral components ranging from 0.2 to 21.4 THz and peak fields exceeding 1.1 kV cm^–1^. Owing to the lattice-matched epitaxial growth, even stronger fields can be realised by increasing the number of QWs. The functionality of our emitter scheme does not require external electric or magnetic biasing, making it easy to use. The tailorable interband resonances make this approach compatible with a broad range of pump lasers. We also see a large potential for further development of our emitters, e.g., by employing non-polar materials, such as silicon and germanium, to avoid lattice absorption, by optimising energy landscapes, and by increasing the in- and outcoupling efficiency via index-matched prisms. Owing to the versatility of the emitter scheme, waveforms, spectra, and field strengths can be adjusted to many applications, such as nonlinear light–matter interaction, ultrabroadband spectroscopy, and femtosecond nanoscopy.

## Materials and methods

### Sample growth

The samples were grown on a semi-insulating (100) GaAs substrate by molecular beam epitaxy. To overcome the lattice-mismatch of 1% between the GaAs substrate and the active layers, an In_*x*_Al_1–*x*_As step-graded buffer was utilised. In this buffer, the indium content was increased from *x* = 0 to 0.15 in three 50 nm steps (*x* = 0.05, 0.10 and 0.15) and a 50 nm step back to *x* = 0.124. The following 100 nm thick constant composition In_0.124_Al_0.876_As layer then serves as a lattice-matched virtual substrate for the following active layers. The buffer was grown at a substrate temperature *T* = 335 °C, while the active layers were grown at *T* = 450–470 °C. The active layers are composed as follows: as described in the main text, InAlAs provides the QW potential walls, while the type-II heterostructure QW is built from a suitable combination of InGaAs and GaAsSb. Each QW was embedded into 30 nm In_0.124_Al_0.876_As on each side. The QW is formed with 7 nm In_0.141_Ga_0.859_As and 7 nm GaAs_0.87_Sb_0.13_. The heterostructures were capped with 2 nm In_0.141_Ga_0.859_As to prevent oxidation damage.

## Supplementary information

Supplementary Information

## Data Availability

The data sets generated and/or analysed during the current study are available from the corresponding authors upon reasonable request.
