# Supplementary material for: Scalable high-repetition-rate sub-half-cycle terahertz pulses from spatially indirect interband transitions
Source: Light Sci Appl. 2022 May 23;11:151. doi: 10.1038/s41377-022-00824-6 (PMC9127092; doi:10.1038/s41377-022-00824-6)
Supplement: Supplementary file 1 — Supplementary Information [file 41377_2022_824_MOESM1_ESM.docx]

**Supplementary Information for
“Scalable high-repetition-rate sub-half-cycle terahertz pulses from spatially indirect interband transitions”**

Christian Meineke^1,^ *^+^*, Michael Prager^1,^ *^+^*, Johannes Hayes^1^, Qiannan Wen^2,^ *^+^*, Lukas Zheyi Kastner^1^, Dieter Schuh^1^, Kilian Fritsch^3^, Oleg Pronin^3^, Markus Stein^4^, Felix Schäfer^4^, Sangam Chatterjee^4^, Mackillo Kira^2, *^, Rupert Huber^1, *^, Dominique Bougeard^1^

*^1^ Department of Physics, University of Regensburg, 93040 Regensburg, Germany
^2^ Department of Electrical Engineering and Computer Science, University of Michigan, Ann Arbor, MI 48109, USA.*

*^3^ Faculty of Electrical Engineering, Helmut Schmidt University, 22043 Hamburg, Germany*

*^4^ Institute of Experimental Physics I, Justus Liebig University Giessen, 35392 Giessen, Germany
^+^ shared first authorship,
^*^ Email: mackkira@umich.edu; rupert.huber@ur.de*

**Supplementary note 1: Modulation spectroscopy of the quantum well emitter**

The interband transitions in our quantum well (QW) structure (Figure S1a) were examined by means of modulation spectroscopy: The QWs are pumped with laser pulses spectrally centred at 1.46 eV, at a pump fluence of 87.5 µJ cm^-2^. The pump-induced change of the absorption, Δα, is then probed via a supercontinuum which is delayed relative to the pump pulse. In addition to the measurement at room temperature, the modulation spectroscopy was also performed at a lattice temperature of 3 K, where the transitions are much less broadened. Figure S1b shows Δα measured at both temperatures at a delay time between pump and probe of 1 ps.


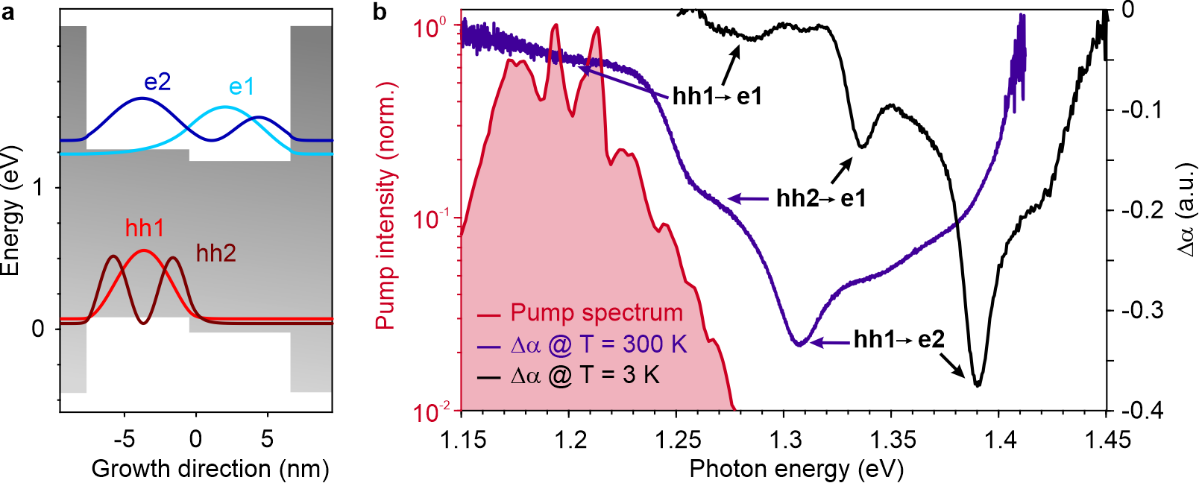


**Figure S1 | Modulation spectroscopy of the quantum well emitter. a**, Quantum well potential of our emitter. The probability densities of the two lowest (highest) electron (heavy-hole) subbands, e1 and e2 (hh1 and hh2) are shown in blue (red). **b**, Pump-induced change of the absorption coefficient, Δα, at 300 K (purple line) and 3 K (black line). At 300 K, the transitions of hh1 and hh2 to e1 are the only transitions that overlap with the pump spectrum (red line).

**Supplementary note 2: Simplistic THz-emission model**

To obtain a first estimate of the shape of the THz transients generated in our QW structures, we employed a simplistic emission model: We assume that by resonant interband excitation, the highest valence and lowest conduction subband are populated. In a static confinement potential, as the envelope functions of the populated subbands are spatially separated, the shift current density *j*(*t*) is proportional to the temporal derivative of the electron density *n_e_*. The latter is generated by the pump intensity *I*_Laser_(*t*) and decays via carrier recombination (recombination time τ). Note, that owing to the quantum confinement in our structures, diffusive currents along the growth direction are not allowed. Thus, we can describe the current along the growth direction of the QWs as

$$j\left( t \right) \sim\frac{dn_{e}(t)}{dt} \sim I_{\mathrm{Laser}}\left( t \right)-\frac{n_{e}\left( t \right)}{\tau}.$$

Owing to the spatial separation of electron and hole wavefunctions, the transition dipole matrix element is small and, hence, large recombination times on the order of nanoseconds are expected. The calculated temporal evolution of the shift currents for a pump pulse with a duration of 140 fs and a recombination time of 1 ns is shown in Figure S2a. The THz far field (Figure S2b) follows the time derivative of the current.


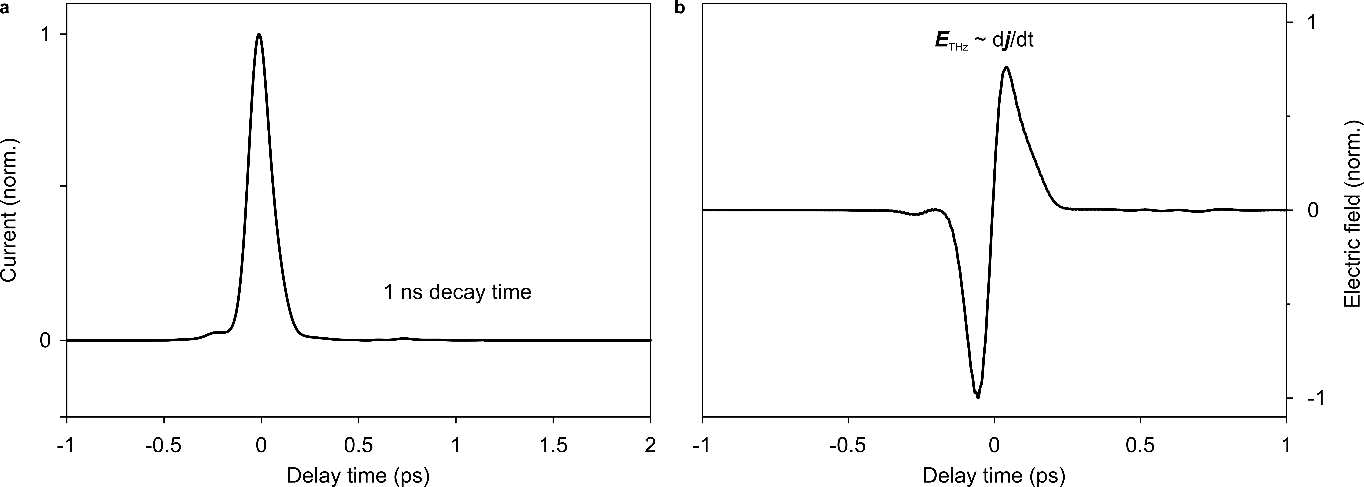


**Figure S2 | Simplistic THz-emission model. a**, Time evolution of the shift-current owing to photoexcitation of spatially separated electron-hole pairs and subsequent recombination with a recombination time of 1 ns. **b**, Corresponding far-field THz waveform.

**Supplementary note 3: Correction for the detector response**

In order to retrieve the actual THz waveform from the measured electro-optic signal, the detector response has to be taken into account. We calculate the detector response function of the employed gallium selenide crystal with a thickness of 6 µm, taking into account the mismatch of the THz phase velocity and group velocity of the gating pulse, absorption in the detector and dispersion of the second-order nonlinearity^1,2^. The complex response function as well as the measured and corrected THz waveform are shown in Figure S3.


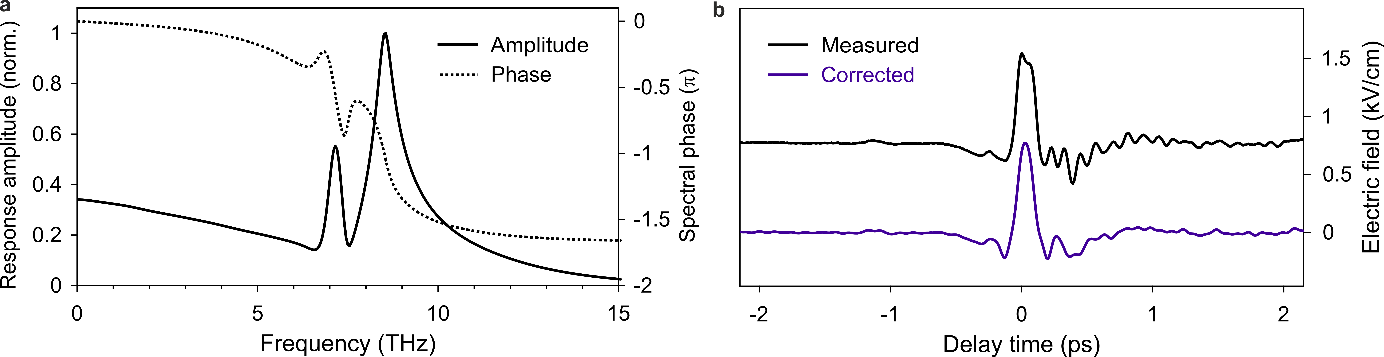


**Figure S3 | Detector response. a**, Response amplitude (solid line) and phase (dotted curve) of the employed GaSe crystal with a thickness of 6 µm in combination with a gate pulse with a duration of 70 fs. **b**, Electro-optic signal (black) and transient corrected for the detector response (purple).

**Supplementary note 4: Determining the number of optical cycles**

The THz transients generated by our QW emitters appear much shorter than an oscillation cycle at their centre frequency. To further quantify this sub-cycle nature, we determine the number of optical cycles within the FWHM of the intensity envelope. To this end, we first determine the field envelope of our waveform: When the carrier-envelope phase is shifted continuously from -π to π the extrema trace the amplitude or field envelope (see Figure S4a). Numerically, one can use the Hilbert transform^3^ *H[..]* to calculate the amplitude envelope *A*(*t*) of the transient electric field *E*(*t*):

$A\left( t \right)= \sqrt{{E(t)}^{2}+{H\left[ E\left( t \right) \right]}^{2}}$.

Furthermore, we calculate the centre frequency *f*_c_ based on energetic weight, i.e.

$$f_{c}=\frac{1}{2\pi}\frac{\int\omega|E\left( \omega\right)|^{2}d\omega}{\int|E\left( \omega\right)|^{2}d\omega},$$

where |*E*(ω)|^2^ is the intensity spectrum of the THz pulse. Now, one can determine how many oscillation cycles with duration T = *f*_c_^-1^ fit into the FWHM of the intensity envelope *I*(*t*) = *A*(*t*)^2^, *t*_FWHM_:

$n_{\mathrm{cycles}}= \frac{t_{\mathrm{FWHM}}}{T}= f_{c}{\times t}_{\mathrm{FWHM}}$.

Note, that the intensity envelope can be well approximated by a Gaussian (see Figure S4b) or sech^2^, such that the use of the FHWM does not lead to arbitrarily small time-bandwidth products. By multiplying the FWHM of the intensity envelope of our pulse (183 fs­) with the FWHM of its spectral amplitude (3.67 THz), we obtain a time-bandwidth product of 0.66.


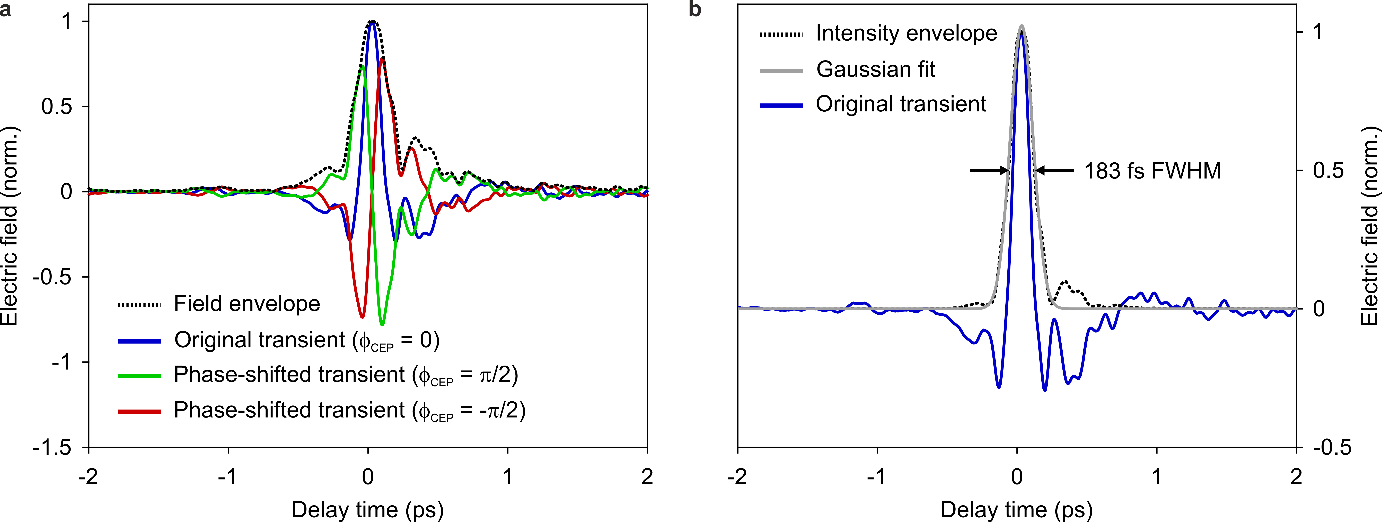


**Figure S4 | Determining the THz pulse envelope. a**, By shifting the carrier-envelope phase φ^­^_CEP_ of the THz waveform (blue, green, red) and tracking the maximal electric-field amplitude at each delay time, an amplitude envelope (black) is received. **b**, The intensity envelope (black dashed line, FWHM 183 fs) is well represented by a Gaussian fit (grey solid line), the FWHM of which is as short as 193 fs.

**Supplementary Note 5: Fine-tuning the asymmetry of the THz waveform**

The asymmetry ratio (i.e. the positive peak field divided by the negative peak field, AR) of our THz transients can be fine-tuned by several parameters: Our theoretical model predicts that the AR can be slightly enhanced via an external bias voltage, which, however, is difficult to implement. A tunnelling barrier between the two QWs reduces the AR as it impedes the local charging dynamics and the unipolar emission it generates. The AR can also be moderately tuned via the pump intensity. According to both our microscopic model and our measurements discussed in the main text, the most effective way to increase the AR is to reduce the pump pulse duration as it accelerates the local charging dynamics, which cause the strong positive oscillation half-cycle of our THz waveforms.

**Supplementary note 6: Determining the focal position of the THz pulses**

In order to avoid artefacts owing to the Gouy phase shift^4^ the electro-optic detector must be placed exactly in the THz focus. We ensure this condition by recording waveforms for different positions of the detector along the beam axis (see Figure S5). The most intense waveform, i.e. the waveform with the smallest beam diameter, coincides with the most asymmetric transient.


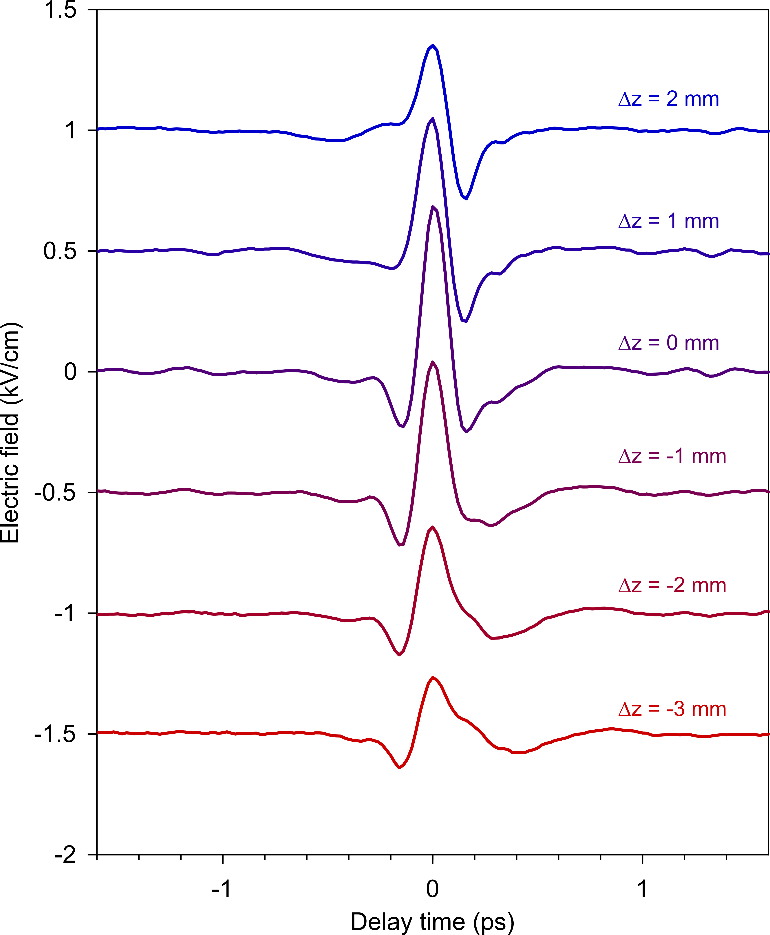


**Figure S5 | Effect of the Gouy phase shift of the THz waveform. a**, THz waveforms measured with a gallium phosphide detector with a thickness of 200 µm, which was moved along the beam axis by a distance Δ*z*. Positive Δ*z* denotes a larger distance between the detector and the focusing mirror. The different waveforms are offset for clarity. At Δ*z* = 0, the asymmetry and intensity of the transient are maximal.

**References**

1. Huber, R., Brodschelm, A., Tauser, F., Leitenstorfer, A. Generation and field-resolved detection of femtosecond electromagnetic pulses tunable up to 41 THz. *Applied Physics Letters* **76**, 3191 (2000).
2. Kampfrath, T., Nötzold, J., Wolf, M. Sampling of broadband terahertz pulses with thick electro-optic crystals. *Applied Physics Letters* **90**, 231113 (2007).
3. Bracewell, R.N. The Fourier Transform And Its Applications. (McGraw Hill, 2000).
4. Feng, S., Winful, H.G. & Hellwarth, R.W. Gouy shift and temporal reshaping of focused single-cycle electromagnetic pulses, *Optics Letters* **23**, 385-387 (1998).
